# Key Factors Correlations in Selecting Dental Services

**DOI:** 10.25122/jml-2019-0023

**Published:** 2019

**Authors:** Ana Maria Cristina Tâncu, Victor Lorin Purcărea, Mihaela Pantea, Marina Imre

**Affiliations:** 1. Department of Complete Denture, Faculty of Dentistry, “Carol Davila” University of Medicine and Pharmacy, Bucharest, Romania; 2. “Carol Davila” University of Medicine and Pharmacy, Bucharest, Romania; 3. Department of Fixed Prosthodontics and Occlusology, Faculty of Dentistry, “Carol Davila” University of Medicine and Pharmacy, Bucharest, Romania

**Keywords:** dental office, patient choice, medical and external factors

## Abstract

The factors which influence patients in their choice of a dental care office has recently become more complex. Our objective was to assess the correlations between different key factors (demographic aspects, dentists’ professionalism, factors related to dental care offices) that influence the selection of a particular dental care office by adult patients.

An online questionnaire (self-administered survey) was applied to a random sample of 117 adult dental patients in private clinics in Bucharest, Romania. The survey consisted of 12 questions, and it was conducted during a 2-week period. All the collected data were subjected to statistical analysis.

The obtained results revealed statistically significant correlations between studied factors, i.e., elderly patients compared to younger patients considered the use of the state-of-the-art medical devices was important for the dental office (p=0.043, R=0.187). In comparison to women, male patients were searching more often information regarding the dental offices on social media (p=0.002, R=0.284); patients that attached more importance to the dentist’s professional degree were the ones that attached more importance to its reputation (p<0.001, R=0.381) and to the dental office location (p=0.022, R=0.211).

The results highlighted specific patterns in patients’ perception of factors related to the selection of dental offices, as also found in scientific literature.

The present study offers a perspective on how to improve dental care and patients’ oral health.

## Introduction

It is still a matter of interest to the population choosing a dentist and a dental office and – sometimes – could be challenging for the patient. Patients choose their dentist based on a variety of factors [1, 2]. Among these, professionalism – a key factor of the doctor-patient relationship – was recognized as a fundamental competency of medical/dental practice in the early years of the twenty-first century [3]. Professionalism involves essential responsibilities, such as commitment to professional competence, honesty with patients, patient confidentiality and maintaining appropriate relations with patients [4]. Taibah ranked the first four most important elements of dental professionalism from patients’ perspective, as follows: good communication skills; humanism and commitment; competence in practice; dentists’ duties and management skills [3]. Previous observations pointed out that most of the patients thought that an empathetic dentist with high dental skills and an updated, hygienic practice are important. Scientific literature offers diverse information on factors influencing patients’ choosing of dental services (Braman, Gomez – 2004; Derose, Hays, McCaffrey, Baker – 2001; Furnham, Petrides, Temple – 2006 – as cited by Furnham et al. [5]. Some patients choose their dentists based on traditional criteria (trust based on a recommendation from family or close friends, dentists’ professional degrees), either based on present-day, modern criteria (dentist’s presence on a particular social network, the webpage of dental office/dentist, online ads) [6]. Therefore, we considered appropriate to analyze the correlations between different categories of factors: patient-related factors (i.e., social-demographic factors, frequency of dental visits , availability for online information), dentist-related factors (clinical competencies, professional degrees, reputation), dental office-related factors (location, costs, hygiene, sterilization and infection control rules, state-of-the-art medical devices), factors associated with the courtesy of the medical staff. Besides, accessibility is important when it comes to choosing a dental care office; therefore, its location is important to the patients, especially considering the heavy traffic in a big city, a situation all our respondents have to deal with [7]. Knowing the factors which influence patients in their choice of a dental care office has recently become far more challenging to dentists. In this context, this study aims to analyze the correlation between patients’ socio-demographic factors and the other previously mentioned factors – that influence the choice of dental care office by the patients. All these aspects have an essential role when it comes to correlating dental services offered to patients with their expectations and offer support for the improvement of dental services.

## Materials and Methods

### Study design

An online questionnaire was elaborated by the authors and distributed to a random group of 117 adult dental patients in private clinics in Bucharest, Romania. The participants were requested to respond to questions about choosing a dental care office. The online (self-administered) questionnaire with the title “The assessment of the criteria influencing the choice of a dentist/dental care office” comprised 12 questions; it was made under the protection of anonymity, as personal information such as first name, surname, personal number was not requested from the persons included in the survey. The questionnaire included two initial questions regarding information on the respondents (age, gender); the questions 3, 4, 5, 7, 8, 9, 10 and 12 were closed multiple-choice questions with the possibility of choosing a single answer, and the questions 6 and 11 were closed multiple-choice questions with the possibility of choosing several answers. The estimated answer time was a maximum of 2 minutes. The questionnaire was created with the aid of the Google Forms software, and it was sent to the persons included in the survey through their email addresses – by a message which included the link of the questionnaire. This questionnaire was chosen thanks to its accessibility, through computer, tablet and smartphone platforms. The questionnaire was filled in online, and it took 14 days to get all the answers. At the end of the period necessary for filling in the questionnaires, these were counted and validated; out of a total of 117 questionnaires, 117 were valid and none was invalidated.

### Data analysis

All collected data were analyzed using IBM SPSS Statistics 20. Quantitative variables were tested for normal distribution using the Shapiro-Wilk Test and were written as averages with standard deviations, and categorical variables were written as counts or percentages. Quantitative variables were tested using Mann-Whitney U tests because of their non-parametric distribution, and all the existing correlations were demonstrated using Spearman’s rho Correlations, while categorical variables were tested using Pearson Chi-Square or Fisher’s Exact tests; all existent correlations were demonstrated using Pearson’s correlation coefficient

## Results

The analyze of collected data corresponding to the participants included in this study reveals that most of them are women (71.8%), with university studies (85.5%) and visit their dentists regularly (74.4%). The data also show that patients are middle-aged (average age is 41.22 ± 13.074 years) and most of the patients learned about their dentist/dental office from family or friends (99.1%); few of them also searched online reviews (20.5%) or social networks (8.5%) for more information, as it can be seen in Table 1.

**Table 1:** Distribution of the demographic data

Demographic data	Average ± SD	Interval
Age	41.22 ± 13.074	18-81
Gender	N	%
Female	84	71.8%
Male	33	28.2%
Education degree	N	%
Secondary education	17	14.5%
Tertiary education	100	85.5%
Reason for check-up	N	%
Only for toothache or emergencies	30	25.6%
Regular check-up	87	74.4%
Source of information	N	%
Direct recommendation from family/friends	116	99.1% (Of total)
Online reviews	24	20.5% (Of total)
Social networks	10	8.5% (Of total)
Mass-media advertising	4	3.4% (Of total)

Further analysis made on the participants’ responses showed the following elements (Table 2):

– most of the patients consider the dentist reputation very important (41.9%) or important (31.6%) for the dental office reputation;
– most of the patients consider the dentist’s professional degree also important (40.2%) or very important (36.8%) for the dental office reputation;
– most of the patients consider the communication availability of the medical staff very important (70.1%) or important (26.5%) for the dental office reputation.

**Table 2:** Analysis of the importance of different aspects considering the dental office/medical staff that may influence the patient’s decision of seeing the dentist regulary

Aspect / Importance	Low N (%)	Medium N (%)	High N (%)	Very High N (%)
Dentist reputation	1 (0.9%)	30 (25.6%)	37 (31.6%)	49 (41.9%)
Professional degree	3 (2.6%)	24 (20.5%)	47 (40.2%)	43 (36.8%)
Location of the dental office	10 (8.5%)	40 (34.2%)	39 (33.3%)	28 (23.9%)
Cost of medical services	4 (3.4%)	52 (44.4%)	42 (35.9%)	19 (16.2%)
Method of payment	29 (24.8%)	39 (33.3%)	29 (24.8%)	20 (17.1%)
Communication availability of the medical staff	0 (0%)	4 (3.4%)	31 (26.5%)	82 (70.1%)

Other studied aspects that may influence the patient’s decision when it comes to choosing a specific dental care office (adherence to sterilization and infection control rules and procedures; use of state-of-the-art medical devices; existence of a website for the dental office; courtesy of the medical staff) highlighted other interesting elements (as seen in Table 3):

– an important percentage of patients consider that the hygiene of the dental office (94%), the cleanliness (84.6%), the usage of state-of-the-art medical devices (81.2%) and the courtesy of the medical staff (79.5%) are essential for the dental office reputation;
– on the other hand, few participants (23.1%) consider that having a website for the dental office is important for the dental office reputation.

**Table 3:** Comparative analysis of aspects related to the adherence to sterilization, the use of state-of-the-art medical devices, and the courtesy of the medical staff and the existence of a website for the dental office

Aspect / Importance	Not important N (%)	Important N (%)
Hygiene and optimal sterilization	7 (6%)	110 (94%)
Cleanliness	18 (15.4%)	99 (84.6%)
Use of state-of-the-art medical devices	22 (18.8%)	95 (81.2%)
Courtesy of medical staff	24 (20.5%)	93 (79.5%)
Existence of a website for the dental office	90 (76.9%)	27 (23.1%)

Table 4 shows the correlation between the average age patient and the use of modern, state-of-the-art medical devices in the dental office, as an indicator-factor for its reputation. Using the Mann-Whitney U test, it has been shown that the patients’ age who consider this factor important or not is significantly different (p=0.044) and the positive low-grade correlation (p=0.043, R=0.187) establishes that patients who consider that the use of the state-of-the-art medical devices in the dental office is important are older than the ones who don’t.

**Table 4:** Average value of the patients’ age according to the use of state-of-the-art medical devices in the dental office as a factor for its reputation

Use of state-of-the-art medical devices	Average ± SD	P-value*
Absent	36.09 ± 10.023	0.044
Present	42.41 ± 13.45	0.043, R=0.187**

* Mann-Whitney U Test

** Spearman’s Rho Correlation Coefficient

Significant differences between the gender of the patients and their opinion on different sources of information or the existence of a website of the dental office as a reputation factor are presented in Table 5. It can be seen that there are some significant differences between the percentages of female/male patients who searched information about the dental office on social media (p=0.005) or mass-media advertising (p=0.006) and those who consider the existence of a website for the dental office as an essential factor for its reputation (p=0.033). The correlations that were found show that more male patients were searching for information about the dental office on social media (p=0.002, R=0.284) /mass-media advertising (p=0.001, R=0.300) and considered important the existence of a website for the dental office (p=0.033, R=0.198) in comparison to women who were informed through other means and did not consider the website important as a reputation factor.

**Table 5:** Details on significant differences between the gender of the patients and their opinion on different sources of information or the existence of a website of the dental office as a reputation factor.

Source of information / Gender	Female N (%)	Male N (%)	P-value*
Other source than social media	81 (75.7%)	26 (24.3%)	0.005
Social media	3 (30%)	7 (70%)	0.002, R=0.284**
Other source than mass-media advertising	84 (74.3%)	29 (25.7%)	0.006
Mass-media advertising	0 (0%)	4 (100%)	0.001, R=0.300**
**Dental office reputation factor: Existence of a web-site / Gender**	**Female N (%)**	**Male N (%)**	P-value***
Absent	69 (76.7%)	21 (23.3%)	0.033
Present	15 (55.6%)	12 (44.4%)	0.033, R=0.198**

* Fisher’s Exact Test

** Pearson Correlation Coefficient

*** Pearson Chi-Square Test

Table 6 shows the significant differences between patients’ reasons for check-ups and sources of information/reputation factors. Data show that there are significant differences between patients who see a dentist for regular check-ups and those who visit only for emergencies, in the following groups: patients who search for information about the dental office online reviews or not (p=0.007), patients who attach a different degree of importance to the prices of services (p=0.017) and patients who consider or not the courtesy of the medical staff as an important factor for the dental office reputation (p=0.011). The correlations that were established show that patients who check more on online reviews attend regular check-ups (p=0.007, R=0.250), as well as those who consider the courtesy important (p=0.011, R=0.235). The prices of medical services are not so crucial for the patients who attend regular check-ups; in comparison, the patients who visit the dentist for emergencies attach more often a high/very high importance to the medical services prices (p=0.044, R= -0.187).

**Table 6:** Observations on significant differences between the patients’ reasons for check-ups and sources of information/reputation factors

Source of information / Reason for check-ups	Toothache/Emergencies N (%)	Regular check-up N (%)	P-value*
Other source than on-line reviews	29 (31.2%)	64 (68.8%)	0.007
On-line reviews	1 (4.2%)	23 (95.8%)	0.007, R=0.250**
**Importance of prices for services / Reason for check-ups**	**Toothache/Emergencies N (%)**	**Regular check-up N (%)**	P-value***
Low	2 (50%)	2 (50%)	
Medium	6 (11.5%)	46 (88.5%)	0.017
High	15 (35.7%)	27 (64.3%)	0.044, R= -0.187**
Very High	7 (36.8%)	12 (63.2%)	
**Dental office reputation factor: Courtesy of medical staff / Reason for check-ups**	**Toothache/Emergencies N (%)**	**Regular check-up N (%)**	P-value***
Absent	11 (45.8%)	13 (54.2%)	0.011
Present	19 (20.4%)	74 (79.6%)	0.011, R=0.235**

* Fisher’s Exact Test

** Pearson’s Correlation Coefficient

*** Pearson’s Chi-Square Test

The comparative analysis between the dentists’ professional degree as a reputation factor and other reputation factors (Table 7) shows significant differences: patients attach significantly different importance to the professional degree according to the importance given to the reputation of the physician (p<0.001) and the dental office location (p=0.015). The performed correlations show that patients that attach a higher importance to the professional degree also attach a higher importance to the reputation of the physician (p<0.001, R=0.381) and to the dental office location (p=0.022, R=0.211); therefore, from patients’ perspectives, these are to be considered important factors for the good reputation of the dental office.

**Table 7:** Observations of significant differences between dentists’ professional degree as a reputation factor and other reputation factors

Reputation of Physician Importance / Professional Degree Importance	Low N (%)	Medium N (%)	High N (%)	Very High N (%)	P-value*
Physician Reputation factor: Low	1 (100%)	0 (0%)	0 (0%)	0 (0%)	
Physician Reputation factor: Medium	0 (0%)	15 (50%)	10 (33.3%)	5 (16.7%)	<0.001
Physician Reputation factor: High	1 (2.7%)	3 (8.1%)	21 (56.8%)	12 (32.4%)	<0.001, R=0.381**
Physician Reputation factor: Very High	1 (2%)	6 (12.2%)	16 (32.7%)	26 (53.1%)	
**Dental Office Location Importance / Professional Degree Importance**	**Low N (%)**	**Medium N (%)**	**High N (%)**	**Very High N (%)**	P-value*
Location factor: Low	1 (10%)	2 (20%)	6 (60%)	1 (10%)	
Location factor: Medium	0 (0%)	10 (25%)	19 (47.5%)	11 (27.5%)	0.015
Location factor: High	2 (5.1%)	5 (12.8%)	18 (46.2%)	14 (35.9%)	0.022, R=0.211**
Location factor: Very High	0 (0%)	7 (25%)	4 (14.3%)	17 (60.7%)	

* Pearson’s Chi-Square Test

** Pearson’s Correlation Coefficient

The correlation between the importance of the physician reputation and the communication availability of the medical staff, show significant differences (Table 8), as Pearson’s Chi-Square test demonstrated (p=0.021). Also, the positive low-grade correlation (p=0.004, R=0.261) shows that patients who attach a higher grade of importance to the reputation of the physician also attach a higher grade of importance to the communication availability of the medical staff from the dental office.

**Table 8:** Observations on significant differences between the importance of the physician reputation and the communication availability of the medical staff

Reputation of Physician Importance / Communication availability Importance	Medium N (%)	High N (%)	Very High N (%)	P-value*
Physician Reputation factor: Low	0 (0%)	1 (100%)	0 (0%)	
Physician Reputation factor: Medium	1 (3.3%)	11 (36.7%)	18 (60%)	0.021
Physician Reputation factor: High	2 (5.4%)	14 (37.8%)	21 (56.8%)	0.004, R=0.261**
Physician Reputation factor: Very High	1 (2%)	5 (10.2%)	43 (87.8%)	

* Pearson’s Chi-Square Test

** Pearson’s Correlation Coefficient

The location of the dental office and the price of medical services (as important factors for patients when it comes to choosing a dental care office) were analyzed, and their correlation shows significant differences (Table 9), as Pearson’s Chi-Square Test demonstrated (p=0.001). The positive low-grade correlation (p=0.002, R=0.280) shows that patients who attach a higher grade of importance to the location of the dental office, also attach a higher grade of importance to the price of medical services.

**Table 9:** Observations on significant differences between the location of the dental office and the price of medical services as important factors when it comes to choosing a dental care office

Location Importance / Price of Services Importance	Low N (%)	Medium N (%)	High N (%)	Very High N (%)	P-value*
Location factor: Low	2 (50%)	1 (25%)	0 (0%)	1 (25%)	
Location factor: Medium	5 (9.6%)	21 (40.4%)	15 (28.8%)	11 (21.2%)	0.001
Location factor: High	3 (7.1%)	14 (33.3%)	20 (47.6%)	5 (11.9%)	0.002, R=0.280**
Location factor: Very High	0 (0%)	4 (21.1%)	4 (21.1%)	11 (57.9%)	

* Pearson’s Chi-Square Test

** Pearson’s Correlation Coefficient

Table 10 shows the significant differences between the method of payment and the price of medical services, as important factors in selecting a dental care office, as shown by the Pearson’s Chi-Square test (p<0.001). The positive moderate grade correlation (p<0.001, R=0.421) shows that patients who attach a higher grade of importance to the method of payment for the medical services, also attach a higher grade of importance to the price of the medical services.

**Table 10:** Observations on significant differences between the method of payment and the price of medical services as important factors for patients when it comes to choosing a dental care office

Method of Payment Importance / Price of Services Importance	Low N (%)	Medium N (%)	High N (%)	Very High N (%)	P-value*
Method factor: Low	2 (50%)	1 (25%)	1 (25%)	0 (0%)	
Method factor: Medium	18 (34.6%)	19 (36.5%)	12 (23.1%)	3 (5.8%)	<0.001
Method factor: High	8 (19%)	15 (35.7%)	13 (31%)	6 (14.3%)	<0.001, R=0.421**
Method factor: Very High	1 (5.3%)	4 (21.1%)	3 (15.8%)	11 (57.9%)	

* Pearson’s Chi-Square Test

** Pearson’s Correlation Coefficient

Significant differences between the percentage of patients who consider important the use of state-of-the-art medical devices and other reputation factors are presented in Table 11. Data show that there are significant differences between the groups that consider important or not the use of state-of-the-art medical devices and the groups of patients who consider important or not the cleanliness of the dental office (p=0.018), or the courtesy of medical staff (p=0.041). The established correlations show that the patients who consider the use of state-of-the-art medical devices as an important factor, also consider that the cleanliness of the dental office is essential (p=0.018, R=0.219), as well as the courtesy of the medical staff (p=0.041, R=0.189).

**Table 11:** Correlations regarding the percentage of patients who consider the use of state-of-the-art medical devices as an important factor

Cleanliness Importance / Usage of state-of-the-art medical devices importance	Not important N (%)	Important N (%)	P-value*
Cleanliness not important	7 (38.9%)	11 (61.1%)	0.018
Cleanliness important	15 (15.2%)	84 (84.8%)	0.018, R=0.219**
**Courtesy of medical staff Importance / Usage of state-of-the-art medical devices importance**	**Not important N (%)**	**Important N (%)**	P-value*
Courtesy not important	8 (33.3%)	16 (66.7%)	0.041
Courtesy important	14 (15.1%)	79 (84.9%)	0.041, R=0.189**

* Pearson’s Chi-Square Test

** Pearson’s Correlation Coefficient

## Discussions

The purpose of this survey was to correlate personal and professional factors as well as other external factors (the location of the office, the office equipment, medical costs, methods of payment and source of information) which determine the patients to choose a certain dental care office. The prevalence of these factors in the context of a potential choice was analyzed as well.

The distribution of the demographic factors associated with the surveyed group is characterized by the following elements: the preponderance of middle-aged women (41) with a high educational level (university) who often go to the dentists for regular dental check-ups/therapies – thus, they are an informed and relevant population from the point of view of information on health care.

In our study, consistent with some scientific literature data [8], the doctor’s reputation is a first criterion for choosing a dental care office, followed closely by a criterion which is less mentioned in other specialized publications, namely the doctor’s professional degree – which was considered important and very important by over 2/3 of our respondents. Besides this professional factor, the next position in the hierarchy of the criteria is occupied by the availability of the medical and auxiliary staff that communicates with patients and the courtesy of the medical staff. The rest of the external factors – the location of the office, the equipment, the medical costs, the payment methods and the source of information – are considered in this order by the patients as important in the choice of a dental care office.

Concerning the source of information, the data obtained in this study show that the direct recommendation by other patients is the most important in choosing a dental care office (99%); these aspects are found in the results of previous research [6].

According to the established correlations, older patients show a higher interest in the equipment of the dental care office with state-of-the-art medical devices, as opposed to younger patients. The human factor – namely the availability for communication and the courtesy of the medical staff – is considered to be as one of the most critical criteria for appreciating the professionalism of the medical team; this fact is also found in other specialized publications [3].

The issues regarding hygiene, cleanliness, sterilization, and equipment with state-of-the-art devices take precedence than the existence of a website for the dental office. In this respect, significant gender-related statistic differences were revealed: the correlations show that male patients who search information on social media, websites or mass media advertising are more numerous (p=0.002, R=0.284) than female patients.

Other significant correlations were in connection with the frequency and the reasons why patients see the dentist. Thus, the data obtained from this survey group show that those patients who search online information on dental care services are those who regularly go for check-ups (p=0.007, R=0.250), as well as the fact that the medical staff’s courtesy is essential (p=0.011, R=0.235); on the other hand, the cost is not a primordial criterion to them. In the case of those patients who go to the dental care office for emergencies, the price criterion is an important one – this fact is also found in other publications [7, 9].

## Conclusions

Professionalism attributes that must be owned by the physician remains the primary criterion when it comes to choosing a specific dental care office by patients – fact asserted by the specialty literature consulted. The study also points out that the most trusted recommendation when it comes to choosing a dentist or dental care office (especially for female patients) is the direct one, contrary to the current trends in online advertising. Adherence to sterilization, infection control rules, and procedures as well as the courtesy of the medical staff are factors that most participants are more respondent to than dental office location or cost of medical treatments. There are significant differences between the criteria of patients who regularly go for check-ups and those who go only occasionally for emergencies, but these criteria are not related to the reputation of the physician but the external factors such as location or medical costs.

Establishing a dental care office or rehabilitating an existing practice are ambitious, complex projects that require much work, dedication and financial efforts from the dentist. The results of this study reveal that professional training is the primary criterion in the success of such projects, but that there are collateral factors that influence patients in choosing a specific dental care office, which should guide the dentist in organizing its work.

## Conflict of Interest

The authors confirm that there are no conflicts of interest.
